# Acute D3 Antagonist GSK598809 Selectively Enhances Neural Response During Monetary Reward Anticipation in Drug and Alcohol Dependence

**DOI:** 10.1038/npp.2016.289

**Published:** 2017-01-25

**Authors:** Anna Murphy, Liam J Nestor, John McGonigle, Louise Paterson, Venkataramana Boyapati, Karen D Ersche, Remy Flechais, Shankar Kuchibatla, Antonio Metastasio, Csaba Orban, Filippo Passetti, Laurence Reed, Dana Smith, John Suckling, Eleanor Taylor, Trevor W Robbins, Anne Lingford-Hughes, David J Nutt, John FW Deakin, Rebecca Elliott, ICCAM Platform

**Affiliations:** 1 Neuroscience and Psychiatry Unit, University of Manchester, Manchester, UK; 2 Centre for Neuropsychopharmacology, Division of Brain Sciences, Imperial College London, London, UK; 3 Department of Psychiatry, University of Cambridge, Cambridge, UK; 4 Department of Psychology, University of Cambridge, Cambridge, UK

## Abstract

Evidence suggests that disturbances in neurobiological mechanisms of reward and inhibitory control maintain addiction and provoke relapse during abstinence. Abnormalities within the dopamine system may contribute to these disturbances and pharmacologically targeting the D3 dopamine receptor (DRD3) is therefore of significant clinical interest. We used functional magnetic resonance imaging to investigate the acute effects of the DRD3 antagonist GSK598809 on anticipatory reward processing, using the monetary incentive delay task (MIDT), and response inhibition using the Go/No-Go task (GNGT). A double-blind, placebo-controlled, crossover design approach was used in abstinent alcohol dependent, abstinent poly-drug dependent and healthy control volunteers. For the MIDT, there was evidence of blunted ventral striatal response to reward in the poly-drug-dependent group under placebo. GSK598809 normalized ventral striatal reward response and enhanced response in the DRD3-rich regions of the ventral pallidum and substantia nigra. Exploratory investigations suggested that the effects of GSK598809 were mainly driven by those with primary dependence on alcohol but not on opiates. Taken together, these findings suggest that GSK598809 may remediate reward deficits in substance dependence. For the GNGT, enhanced response in the inferior frontal cortex of the poly-drug group was found. However, there were no effects of GSK598809 on the neural network underlying response inhibition nor were there any behavioral drug effects on response inhibition. GSK598809 modulated the neural network underlying reward anticipation but not response inhibition, suggesting that DRD3 antagonists may restore reward deficits in addiction.

## INTRODUCTION

Evidence suggests dysregulation of neurobiological networks involved in reward processing and inhibitory control contributes to the risk and maintenance of addiction and relapse during abstinence. Disturbances in reward functioning involve hyporesponsivity to non-drug reward, which is associated with increased craving, drug use, and brain response to drug-related stimuli (Blum *et al*, 2000; Lubman *et al*, 2009; Wrase *et al*, 2007). Failures of impulse control across a range of domains have been a consistent finding in addiction and are associated with relapse (Taylor *et al*, 2016). fMRI studies implicate reduced recruitment of lateral and medial prefrontal regions in impaired impulse control (Forman *et al*, 2004; Kaufman *et al*, 2003), although enhanced recruitment has been found in those who have successfully achieved abstinence from cocaine (Connolly *et al*, 2012).

Abnormalities in reward and impulse control may be effects of blunted dopamine signaling in addiction (Trifilieff and Martinez, 2014). Reductions in dopamine release and receptor density are associated with increased drug use and craving and may precede the development of addiction (Casey *et al*, 2014; Heinz *et al*, 2004). Dopamine has a pivotal role in reward-related behaviors, mediating reward learning (Schultz, 1998) and ‘incentive salience’ of reward stimuli (Robinson and Berridge, 1993). Deficits in dopamine neurotransmission may impair impulse control, as low striatal D2/D3 binding is associated with increased impulsivity in rodents and humans (Clark *et al*, 2012; Ghahremani *et al*, 2012). Drugs increasing extracellular dopamine improve response inhibition in cocaine and alcohol dependence (Garrison and Potenza, 2014). These lines of evidence suggest that increasing brain dopamine may be a useful therapeutic strategy for addiction (Nutt *et al*, 2015).

The D3 receptor (DRD3) is preferentially expressed within ventral striatal and limbic brain regions involved in reward processing. *In vitro* studies demonstrate the highest density of DRD3s within the ventral striatum of the human brain (Gurevich and Joyce, 1999), whereas *in vivo* human positron emission tomography (PET) studies demonstrated maximal DRD3 density within the ventral pallidum (VP), followed by the substantia nigra (SN) and ventral striatum (VS), with lower levels in thalamus and dorsal striatum (Tziortzi *et al*, 2011). Exposure to drugs of abuse results in upregulation of DRD3 in rodent models of addiction (Le Foll and Di Ciano, 2015). Upregulated DRD3 density has been found within the VS and SN in a postmortem study of cocaine dependence (Staley and Mash, 1996) while upregulated nigral DRD3s correlated positively with impulsivity and risky decision making in stimulant dependence (Boileau *et al*, 2012; Payer *et al*, 2014). Trends for upregulated ventral pallidal DRD3 have been found in both alcohol dependence and heavy stimulant use (Boileau *et al*, 2012; Erritzoe *et al*, 2014). Consequently, there is much interest in this receptor as a target for drug therapy.

DRD3 antagonists have shown promise in preclinical studies, reducing self-administration, cue-induced drug-seeking, and conditioned place preference (Heidbreder and Newman, 2010). In clinical populations, the novel DRD3 antagonist GSK598809 transiently reduced craving in nicotine dependence (Mugnaini *et al*, 2013) and attentional bias for food cues in low restrained eaters (Nathan *et al*, 2012). The exact mechanism by which DRD3 antagonists achieve these effects is currently unknown, although there is evidence that the DRD3 is an autoreceptor controlling the synthesis and release of dopamine (Diaz *et al*, 2000; Zapata and Shippenberg, 2002). Blockade of DRD3 with GSK598809 may therefore increase extra-synaptic dopamine.

The ICCAM Platform study is a multicenter research study that aimed to (1) identify brain networks underlying addiction to alcohol, cocaine, and opiates and relapse vulnerability and (2) identify potential new treatments for addiction based on their ability to modulate these networks. Here we present the effects of GSK598809 on networks underlying the anticipation of reward, using the monetary incentive delay task (MIDT; Knutson *et al*, 2000) and response inhibition using the Go/No-Go task (GNGT; Garavan *et al*, 2002). We hypothesized that reward and inhibitory control disturbances would be found in abstinent drug-dependent individuals and that GSK598809 would mitigate such disturbances.

## MATERIALS AND METHODS

### Participants

Participants were recruited as part of the ICCAM multicenter study. Detailed description of recruitment and participant characteristics are described elsewhere (Paterson *et al*, 2015). Briefly, substance-dependent individuals were recruited according to the following criteria: aged 20–64 years, meeting DSM-IV (American Psychiatric Association, 2000) criteria for current or prior alcohol, cocaine or opiate dependence, abstinent for at least 4 weeks, free from any current primary axis-1 mental health disorder, no history of severe enduring mental illness, no psychoactive medications, no serious physical health problems, no neurological disease, and no contraindications for MRI scanning. Healthy controls (HC) were recruited according to the same criteria except that they had no current or history of dependence to any drug except nicotine.

Eighty-eight participants completed both placebo and GSK598809 sessions: a HC group (*n*=35), an abstinent alcohol-dependent (AD) (*n*=20) and an abstinent poly-drug-dependent (PD) group (*n*=33). Five people were excluded from the MID analysis, and 14 from the GNG analysis leaving final *N*s of 83 and 74, respectively (see Supplementary Materials for details).

The HC group was matched with the AD and PD groups for age, sex, smoking status, and handedness and additionally with the AD group, but not the PD group, for IQ and years of education (see Supplementary Tables S1 and S2). AD and PD groups differed significantly for age for the MID analysis, with a trend for a difference for the GNG analysis (*p*=0.06).

### Procedures and Tasks

Procedures are described in detail elsewhere (Paterson *et al*, 2015). Briefly, the ICCAM study involved five separate scanning sessions (one screening, including fMRI scanning to familiarize participants with the scanner environment and tasks, and four drug testing sessions). Placebo and GSK598809 (60 mg) were administered in a double-blind manner with a crossover design. Owing to concerns over study dropout and loss of placebo data, a weighted randomization was used with the placebo session administered in study session 2 or 3, whereas GSK598809 was administered in session 4 or 5 (with the other two sessions testing other drugs as part of the ICCAM platform; Paterson *et al*, 2015). The mean (SD) inter-session interval between placebo and GSK598809 sessions was 34.39 days (40.91) (MIDT) and 36.15 days (42.72) (GNGT), with no difference between groups; MID (F(2.80)=0.25, *p*=0.78), GNG (F(2,71)=0.03, *p*=0.98).

Scans occurred 2 h after administration of drug or placebo and tasks were practiced before scanning. All participants had an alcohol breathalyzer reading of 0.0%. Participants were urine screened. A positive test for cannabis was allowed owing to its long half-life provided there was no use in the previous week. Participants tested negative for all other drugs, with two allowed exceptions (see Supplementary Materials).

### Tasks

Both the MIDT and GNGT are described in detail within Supplementary Materials.

The MIDT was modified from Knutson *et al* (2000) and was designed to probe reward sensitivity. Participants could win or lose money (or neither win nor lose) depending upon how quickly they reacted to a target stimulus that was predicted by a win, loss, or neutral cue. The task was designed such that win accuracy would be 66% and £10 would be won at each session.

The GNGT was an event-related task adapted from Garavan *et al* (2002), consisting of a series of letter Xs and letter Ys. Participants were instructed to respond as fast as they could to each letter (Go trial) except when the letter was the same as the previous letter (No-Go trial).

### Analysis of Behavioral Data

For the MIDT, reward-neutral reaction time (RT) was analyzed. For the GNGT, percentage accuracy for Go trials and No-Go trials and RT for Go trials were analyzed. All analyses used mixed ANOVAs with drug session as the within-subject factor and group as the between-subject factor. Age was included as a mean-adjusted covariate in all analyses.

### Analysis of fMRI Data

Details of data acquisition and preprocessing are in Supplementary Materials.

For the MIDT, analysis focused on the ‘cue and anticipation’ phase and was modeled as a mini-block beginning at the cue onset and ending at the onset of the target stimulus (see Supplementary Materials for details). The contrast of interest is the average of the ‘reward cue anticipation’ compared with ‘neutral cue anticipation’ across both runs.

For the GNGT, successful inhibitions of No-Go trials (‘stops’) and unsuccessful No-Gos (‘errors’) were modeled against an implicit baseline of Go trials. Stops that were preceded by a Go trial that also did not have a response were considered ‘fake inhibitions’ and were modeled separately as conditions of no-interest. The task was powered to look at ‘stops’ rather than ‘errors’, therefore only the ‘stops>go’ contrast is explored further.

Realignment parameters and movement outliers (scan-to-scan displacement of >3 mm) were added to the models as nuisance regressors.

A region of interest (ROI) approach was used. ROIs of the VS, VP, and SN were chosen for the MIDT owing to their key roles in reward processing (Haber and Knutson, 2010) and evidence of abnormalities within these regions in addiction. Additionally, as reviewed above, these regions are particularly rich in DRD3s and therefore are potential targets for GSK598809 effects. For the GNGT, bilateral inferior frontal gyri (IFG) and anterior cingulate cortex were chosen owing to their key role in motor inhibition (Aron *et al*, 2003) and abnormal recruitment in addiction. While not key ROIs for this task, exploratory investigations were also carried out with the DRD3-rich regions of VS, VP, and SN.

**Figure 1:**
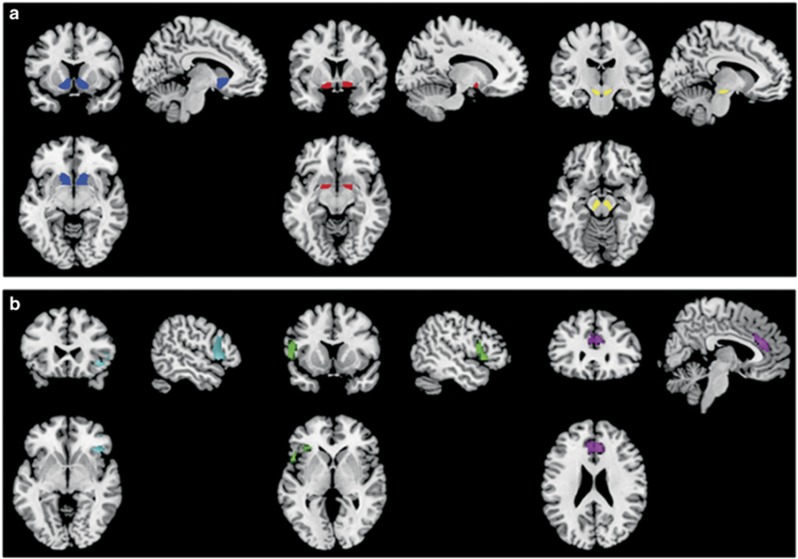
Regions of interest: (a) ROIs for the MID task, left/blue (when in color) shows the ventral striatum, middle/red shows the ventral pallidum (both defined according to the guidelines of Tziortzi *et al*, 2011), and right/yellow shows the substantia nigra. (b) ROIs for the GNG task, left/cyan shows the right inferior frontal gyrus, middle/green shows the left inferior frontal gyrus, and right/purple shows the anterior cingulate.

Mean reward-neutral (MID) and stop-go (GNG) contrast estimates were extracted from the relevant ROIs for each participant and entered into mixed ANOVAs (see Supplementary Materials for details). A Bonferroni’s correction for the three regions investigated for each task was applied, with significance set at *p*<0.017. Additional exploratory whole-brain investigations were carried out using a voxel-wise threshold of *p*<0.05, Family-Wise Error Corrected (see Figure 1 for ROIs and Supplementary Materials for further details).

Correlational analyses were additionally carried out to investigate the relationships between ROI brain response and performance, reward and impulsivity variables, ROI brain response and duration of abstinence, and drug effects on ROI response and performance (see Supplementary Materials for details). Twenty-nine analyses were carried out and Bonferroni-corrected significance was set at *p*<0.0017.

## RESULTS

### MID Behavioral

There were no significant drug or group effects or interactions for MID performance (see Supplementary Figure S1).

### MID fMRI

#### Effect of task

The reward-neutral contrast for each group for both the placebo and GSK598809 conditions revealed a highly significant network of activation, including the VP, VS, and SN, in line with previous studies (Knutson *et al*, 2000). See Supplementary Figure S3.

#### Effects of group and drug: whole-brain analysis

Mixed ANOVAs demonstrated a significant main effect of drug within the left VP, caudate, and cerebellum (Table 1 and Supplementary Figure S5). These effects appear to be driven by increased reward-neutral anticipation response in the GSK598809 session compared with the placebo session, in particular for the AD group and to a lesser extent the PD group. However, these apparent interactions were not significant.

A significant effect of drug was also observed in the right middle frontal gyrus (corresponding to the dorsolateral prefrontal cortex (DLPFC), see Table 1 and Supplementary Figure S3), which was driven by a significant group-by-drug session interaction. GSK598809 increases reward-neutral BOLD response to a greater degree in the AD group compared with both the HC and PD groups.

#### ROI analysis

**Figure 2:**
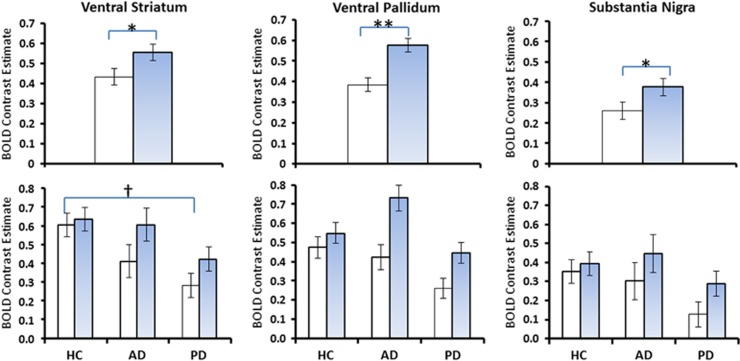
ROI response during the MID task: mean reward-neutral anticipation BOLD contrast estimate for both the placebo and the GSK598809 sessions. White bars represent the placebo session, whereas light gray/blue (when in color) represent the GSK598809 session. Histograms on the top show the main effect of drug within each ROI (*p<0.01, **p<0.001), whereas histograms below show the BOLD contrast estimates for the placebo and GSK598809 sessions for each group separately (†significant effect of group at *p*<0.01 in the placebo condition only). Error bars indicate within-subject SEM (Cousineau and O’Brien, 2014) suitable for assessing drug rather than group effects. A full color version of this figure is available at the *Neuropsychopharmacology* journal online.

Mixed ANOVAs demonstrated a main effect of drug within the VS (*p*=0.005), VP (*p*<0.001), and SN (*p*=0.009) (Table 1). These effects are due to increased reward-neutral BOLD response in the GSK598809 session compared with the placebo session (Figure 2). Although these effects appear to be mainly driven by the dependent groups in each ROI, only a trend for a drug-by-group interaction was found and only in the VP (*p*=0.041, Table 1). *Post hoc* paired *t*-tests revealed a significant effect of GSK598809 on VP reward-neutral BOLD response within the AD group (*p*<0.001) and PD group (*p*=0.003) but not the HC group (*p*=0.145).

No significant main effects of group were found although trends were found within the VS and SN (*p*=0.048 and 0.042, respectively). Figure 2 suggests that these effects were driven by blunting occurring within the placebo condition of the PD group. Exploratory *post hoc* investigations carried out with the placebo session data only demonstrated a significant main effect of group in the VS (F(2,79)=5.03, *p*=0.009, PD<HC) and a trend for a significant blunting in the SN that just fell short of Bonferroni-corrected significance, (F(2,79)=5.03, *p*=0.022, PD<HC). *Post hoc* tests revealed no difference or trends between AD and HC or AD and PD. No group effects or trends emerged for corresponding analysis of the GSK598809 session.

Additional exploratory investigations were carried out within the ROIs, separating the groups by primary drug of dependence (see Supplementary Materials). This suggested that drug effects were driven by participants with a primary alcohol but not opiate dependence (see Supplementary Figure S7). Investigations into primary cocaine dependence were not carried out owing to small numbers.

### GNG Behavioral

There were no significant drug or group effects or interactions for GNG performance (see Supplementary Figure S1).

### GNG fMRI—Effect of Task

The stops>go contrast for each group for both the placebo and GSK598809 conditions revealed a highly significant network of activation, in line with previous studies using this task (Garavan *et al*, 2002). See Supplementary Figure S4.

#### Effects of group and drug: whole-brain analysis

Whole-brain analyses using mixed ANOVAs revealed a significant group effect within the left cerebral peduncle region of the midbrain owing to increased activation in the AD group (Supplementary Figure S6). No drug effects or interactions were found.

#### ROI analysis

**Figure 3:**
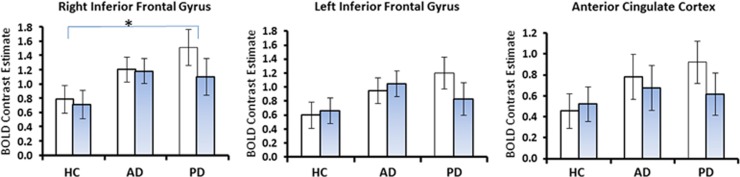
ROI response during the GNG task: mean ‘stops-go’ BOLD contrast estimate within each ROI for the placebo (white bars) and the GSK598809 (gray or blue when in color) sessions. Error bars indicate within-subject SEM (Cousineau and O’Brien, 2014). *Main effect of group, significant at a Bonferroni corrected value of *p*<0.017. A full color version of this figure is available at the *Neuropsychopharmacology* journal online.

There was no effect of GSK598809 in any of the ROIs. A main effect of group was found in the right IFG (F(2,)=4.71, *p*=0.012), and at trend level in the left IFG (p=0.024), driven by hyperactivation in the PD compared with the HC group (*p*=0.016). See Figure 3.

Exploratory analysis revealed no significant effects of GSK598809 in DRD3-rich regions (VS, VP, SN).

### Correlational Analyses

Ventral striatal ROI activation during the placebo condition for the MIDT, in all participants, correlated with impulsivity measured by the Barratt Impulsivity Scale (BIS) (Patton *et al*, 1995) (*r*=−0.368, *p*=0.001) but not the reward responsivity subscale of the BISBAS (Carver and White, 1994) (Supplementary Table S4). To rule out the possibility of this negative association being primarily driven by group differences, an additional correlation was carried out within the healthy control group only, which supported this negative relationship (*r*=−0.372, *p*=0.033). No correlations were found with any of the GNG ROIs (Supplementary Table S3 and S4). No relationships were found between the MID and GNGT ROI activation or fMRI and questionnaire behavioral measures (Supplementary Table S4) or with duration of abstinence (Supplementary Table S5). No relationship was found between the effect of GSK598809 on VP activation and MID performance (Supplementary Table S6).

## DISCUSSION

This study aimed to investigate the effects of the selective DRD3 antagonist GSK598809 on networks involved in the anticipation of reward and response inhibition. The main findings were that GSK598809 significantly increased reward-neutral reward anticipatory responses for the MIDT but had no effects on brain response during response inhibition.

Group effects were found for both tasks, although in opposite directions. Blunting of the VS in the PD group and DLPFC response in the AD group was found for the MIDT under placebo. Enhanced inferior frontal response in the PD group and enhanced midbrain response in the AD group was found for the GNGT. There were no effects of either drug or group on task performance, therefore all reported differences in BOLD response occur in the context of normal performance.

### MID

Deficits in ventral striatal reward-related signaling are hypothesized to confer vulnerability to addiction and relapse (Trifilieff and Martinez, 2014; Blum *et al*, 2000). We found blunted ventral striatal response in the PD group, and an overall negative correlation between VS response and impulsivity. Reduced MID VS response associated with increased impulsivity has been previously demonstrated in alcohol dependence (Beck *et al*, 2009; Wrase *et al*, 2007), therefore our finding adds to a growing literature supporting the reward deficiency hypothesis of addiction. In contrast to the studies above, we did not find significant blunting in the AD group. This could suggest recovery with long-term abstinence in our cohort; however, we did not find a significant association between the length of abstinence and ventral striatal response in the AD group, perhaps owing to small sample size.

There was a general restorative or enhancing effect of GSK598809 on reward anticipatory BOLD response. This finding is in line with evidence suggesting that D3 receptors may act as autoreceptors, inhibiting dopamine synthesis and release (Diaz *et al*, 2000; Zapata and Shippenberg, 2002). It has been hypothesized that tonic extracellular dopamine may inhibit phasic dopamine reward signaling via actions on autoreceptors (Grace, 1991). This may explain the opposing effects on MID reward anticipatory BOLD response of GSK598809 and amphetamine, which releases dopamine. Amphetamine increases tonic dopamine levels but decreasesreward anticipatory BOLD response (Knutson *et al*, 2004). In contrast, by blocking D3 autoreceptors, GSK598809 may enhance phasic dopamine reward signaling (Sokoloff *et al*, 2006) and reward anticipatory BOLD response. This reward enhancing effect of dopamine autoreceptor blockade is supported by a recent study demonstrating low doses of the D2/D3 antagonist amisulpride (chosen to result in preferential autoreceptor blockade) increased MID reward responsivity in depressed patients (Admon *et al*, 2016).

Despite these BOLD enhancing effects of GSK598809, no behavioral effects were found. This may reflect ceiling effects as the behavioral requirements of the MIDT are very simple—reaction times were unimpaired in the placebo condition, suggesting that participants were already performing at maximum capacity for a speeded motor response. Furthermore, 60 mg of GSK598809 results in only partial D3 blockade (Erritzoe *et al*, 2014), therefore behavioral effects may emerge with increasing dose.

Although no significant drug-by-group interactions were found, the effects of GSK598809 appeared to be largely driven by effects within the abstinent drug-dependent groups rather than controls (especially within the DRD3-rich VP where a strong trend to an interaction was observed). These patterns may be due to upregulation of DRD3s in stimulant and alcohol dependence (Boileau *et al*, 2012; Erritzoe *et al*, 2014; Payer *et al*, 2014; Staley and Mash, 1996) and evidence of enhanced dopamine autoreceptor actions in response to chronic exposure to alcohol (Siciliano *et al*, 2016). While requiring replication in a larger sample, exploratory investigations raised the intriguing possibility that GSK598809 may be relatively ineffective in opiate dependence. At the time of writing, we were unable to find any published studies on DRD3 density in human opiate addicts. However, in contrast to the increases in DRD3 expression in rodents after alcohol and cocaine exposure (Le Foll and Di Ciano, 2015), a very recent study demonstrated that heroin exposure decreased DRD3 expression (Zhu *et al*, 2016), possibly explaining the apparent reduced efficacy of GSK598809 in those with a primary opiate dependence.

In addition to effects within DRD3-rich regions, whole-brain analyses revealed effects within the caudate, cerebellum, and DLPFC. *In vivo* PET studies do not report high DRD3 levels within these regions. An enhanced MIDT reward anticipatory caudate response was also found with DRD3 receptor blocking doses of amisulpride (Admon *et al*, 2016). There is evidence supporting the caudate, lateral prefrontal regions, and cerebellum to be important regions mediating the integration of motivation with goal-directed behavior (Harsay *et al*, 2011; Schutter, 2013; Watanabe and Sakagami, 2007). Enhanced activation within these regions may therefore be a downstream consequence of GSK598809’s effects on the VS and VP, regions critically involved in incentive motivation (Haber and Knutson, 2010; Smith *et al*, 2009).

Together, these findings suggest hypofunctioning of reward signaling in substance dependence. Evidence suggests, in alcohol and stimulant dependence, that this may be caused, in part, by excessive D3 autoreceptor inhibition of dopamine systems. GSK898809 may ameliorate reward deficits by disinhibiting these systems.

### GNG

In contrast to GNG studies demonstrating prefrontal hypoactivation and impaired response inhibition in current dependence, we found hyperactivation of lateral prefrontal regions (PD group) or midbrain (AD group) together with unimpaired behavioral performance. Our results are consistent with two other studies in cocaine and alcohol abstinence (Bell *et al*, 2014; Connolly *et al*, 2012). They perhaps reflect recovery of prefrontal structure and function in successful abstinence owing to cessation of drug use and cognitive control practices (Garavan *et al*, 2013). Whether this hyperactivation, which is also found in relatives of alcohol patients, is protective for relapse or an addiction vulnerability marker is unknown. However, fMRI hyperactivation during cognitive tasks has been reported in detoxified alcohol-dependent participants who subsequently abstained but not in those who relapsed (Charlet *et al*, 2014), supporting the notion that prefrontal hyperactivation may be protective.

Despite previous studies suggesting a link between overexpression of DRD3s and increased impulsivity as measured by questionnaire (Boileau *et al*, 2012; Payer *et al*, 2014), we found no evidence to suggest GSK598809-modulated performance or neuronal response of the network underlying response inhibition. This may be due to the multifaceted nature of impulsivity, with DRD3s affecting some impulsivity measures but not response inhibition as measured here. We found no association between a questionnaire measure of impulsivity and GNG brain response or performance. Furthermore, while mood-related impulsivity correlates more strongly with D2/D3 binding in the ventral rather than dorsal striatum in pathological gamblers (Clark *et al*, 2012), response inhibition correlates with receptor binding in the dorsal (where DRD3 density is low) but not the VS (Ghahremani *et al*, 2012). These findings suggest that different impulsivity measures have different underlying neuro-circuitry. Prefrontal regions are important for response inhibition (Aron *et al*, 2003; Garavan *et al*, 2013), again regions where DRD3s are low. Low DRD3 density in regions implicated in response inhibition may explain the lack of a modulatory effect of GSK598809 in our study. We additionally carried out exploratory investigations within the DRD3-rich regions for the GNGT. However, no effects of GSK598809 modulations were found. These findings suggest that D3 agents selectively modulate brain mechanisms of incentive motivation.

### Limitations

The main limitation of the study was the introduction of an order confound, arising from (ultimately groundless) concerns over study dropout. However, practice and habituation effects were minimized by all participants having carried out the tasks in full within the scanner at screening (placebo session was either the second or third task scanning session), and the tasks being practiced outside the scanner before each session. A *post hoc* exploratory ROI analysis was carried out within the anterior insula, a region considered to process salience and therefore likely to be sensitive to habituation effects. Notably, the anterior insula is reliably implicated in MID performance (in a meta-analysis performed by our group) but is devoid of DRD3s. No effects of GSK598809 or interactions were found within this region, supporting the suggestion that the effects seen were indeed drug effects rather than non-specific habituation effects. Another limitation is the age difference between the AD and PD groups (consistent with typical clinical presentation), therefore caution should be used when interpreting AD *vs* PD differences.

## CONCLUSION

GSK598809 enhances reward anticipatory BOLD response to non-drug rewards within abstinent substance-dependent groups, with strongest effects in those with a primary alcohol dependence. These results have implications for considering D3 antagonism as a potential treatment for normalizing reward deficiencies in substance dependence.

## Funding and disclosure

We thank the Medical Research Council (MRC) for funding the study and GlaxoSmithKline (GSK) for supplying the study drug and funding scans at London. This article presents independent research funded by the Medical Research Council as part of their addiction initiative (grant number G1000018). GSK supplied the GSK598809 drug used in this study and funded the functional and structural MRI scans that took place at Imperial College. David Nutt is an advisor to British National Formulary, MRC, GMC, Department of Health, is President of the European Brain Council, past President of the British Neuroscience Association and European College of Neuropsychopharmacology, chair of DrugScience, is a member of the International Centre for Science in Drug Policy, advisor to Swedish government on drug, alcohol and tobacco research, editor of the Journal of Psychopharmacology, sits on advisory Boards at Actelion MSD, and Nalpharm, has received speaking honoraria (in addition to above) from BMS/Otsuka, GSK, Lilly, and Janssen, Servier, is a member of the Lundbeck International Neuroscience Foundation, has received grants or clinical trial payments from P1vital, MRC, NHS, Lundbeck, has share options with P1vital, has been expert witness in a number of legal cases relating to psychotropic drugs, and has edited/written 30 books—some purchased by pharma companies. Trevor W Robbins has research grants with Eli Lilly and Lundbeck, has received royalties from Cambridge Cognition (CANTAB), has received editorial honoraria from Springer Verlag, Elsevier, and Society for Neuroscience; has performed educational lectures for Merck, Sharpe, and Dohme, and does consultancy work for Cambridge Cognition, Eli Lilly, Lundbeck, Teva, and Shire Pharmaceuticals. Bill Deakin currently advises or carries out research funded by Autifony, Sunovion, Lundbeck, AstraZeneca, and Servier. All payment is to the University of Manchester. Anne Lingford-Hughes has received speaking honoraria and research support from Lundbeck and GSK. Liam J Nestor was a Senior Research Scientist employed by GSK during ICCAM data collection. The other authors declare no conflict of interest.

## Figures and Tables

**Table 1:** Results from ROIs and Whole-brain Analyses for the MID (Top Two Subtables) and GNG (Bottom Two Subtables) Tasks

**MID ROI analysis**			
**Region**	**Effect of drug**	**Group × drug interaction**	**Effect of group**
Ventral striatum	F(1,79)=8.35, *p*=0.005, GSK>PBO	F(2,79)=1.31, *p*=0.28, NS	F(2,79)=3.16, *p*=0.048, NS
Ventral pallidum	F(1,79)=30.83, *p*<0.001, GSK>PBO	F(2,79)=3.32, *p*=0.041, NS	F(2,79)=2.01, *p*=0.141, NS
Substantia nigra	F(1,79)=7.28, *p*=0.009, GSK>BPO	F(2,79)=0.86, *p*=0.43, NS	F(2,79)=3.29, *p*=0.042, NS

**MID whole brain**						
**Contrast**	**Region**	**Cluster size**	**MNI coordinates**	**F-value**	***p*-Value (FWE corrected)**	**Direction**
Main effect of the drug	Ventral pallidum	11	−15,5,−10	34.77	0.001	GSK>Plac
	Middle frontal gyrus	11	30,29,29	29.22	0.003	GSK>Plac
	Right cerebellum	9	39,−49,−28	27.81	0.007	GSK>Plac
	Caudate	19	−15,−1,20	26.51	0.01	GSK>Plac
Drug session × group	Middle frontal gyrus	6	30,32,32	29.22	0.003	GSK>PBO, AD>HC, PD
Main effect of the group	No significant clusters					

**GNG ROI analysis**			
**Region**	**Effect of drug**	**Group × drug interaction**	**Effect of group**
Right inferior frontal gyrus	F(1,70)=1.66, *p*=0.20, NS	F(2,70)=0.88, *p*=0.42, NS	F(2,70)=4.45,*p*=0.015, PD>HC
Left inferior frontal gyrus	F(1,70)=0.35, *p*<0.56, NS	F(2,70)=1.12, *p*=0.29, NS	F(2,70)=3.49, *p*=0.036, NS
Anterior cingulate cortex	F(1,70)=0.99, *p*=0.32, NS	F(2,70)=1.02, *p*=0.37, NS	F(2,70)=3.29, *p*=0.119, NS

**GNG whole brain**						
**Contrast**	**Region**	**Cluster size**	**MNI coordinates**	**F-value**	***p*-Value (FWE corrected)**	**Direction**
Main effect of the drug	No significant clusters					
Drug session × group	No significant clusters					
Main effect of the group	Midbrain	13	15,−7,−10	19.13	0.003	AD>HC,PD
