# Supplementary material for: Acute D3 Antagonist GSK598809 Selectively Enhances Neural Response During Monetary Reward Anticipation in Drug and Alcohol Dependence
Source: Neuropsychopharmacology. 2017 Jan 25;42(5):1049–57. doi: 10.1038/npp.2016.289 (PMC5423526; doi:10.1038/npp.2016.289)
Supplement: Supplementary Information [file npp2016289x1.doc]

**Supplementary Materials**

Participants

|  | Healthy Control n=33 | Abstinent Alcohol dependent n=18 | Abstinent Poly-drug dependent  n=32 | Comparisons |
| --- | --- | --- | --- | --- |
| Age | 42.0 (8.6) | 45.4 (8.1) | 38.4 (7.9) | F=4.25, df 2,80, *p*=0.018, P<A |
| Sex (% male) | 81.8 | 77.8 | 78.1 | X^2^=, p=0.91, ns |
| Smoking Status (% Smoker) | 57.6 | 66.7 | 78.1 | X^2^=3.13, *p*=0.21, ns |
| IQ | 105.8 (10.3) | 104.7 (8.1) | 98.5 (11.6) | F=4.39, df 2,80, *p*=0.015 H>P |
| Years of Education | 13.4 (2.7) | 12.3 (2.9) | 11.0 (2.1) | F=6.72, df 2,80, *p*=0.002 H>P |
| Edinburgh Handedness | 43.4 (58.4) | 50.2 (68.2) | 61.7 (58.3) | F=0.76, df 2,80, *p*=0.47, ns |
| Months Abstinence | - | 14.5 (19.8) | 21.9 (34.7) | t=-0.8, df 48, *p*=0.41, ns |
|  |  |  |  |  |
| Table S1. Demographics of the participants included in the Monetary Incentive Delay task analysis. One way ANOVA and Sidak post-hoc tests were used to compare continuous demographic variables. Chi-squared tests were used for categorical variables. A 2-sample t-test was used to test for differences in months of abstinence between abstinent alcohol and poly-drug dependent groups. Means and standard deviation (in brackets) shown for continuous variables. Abstinence from the main drug of dependence was used for the poly-drug group. |  |  |  |  |

|  | Healthy Control n=31 | Abstinent Alcohol dependent  n=18 | Abstinent Poly-drug dependent  N=25 | Comparisons | |
| --- | --- | --- | --- | --- | --- |
| Age | 40.7 (9.2) | 45.1 (9.5) | 38.5 (8.7) | | F=2.75, df 2,71, *p*=0.07, ns |
| Sex (% male) | 81.8 | 77.8 | 78.1 | | X^2^=, p=0.91, ns |
| Smoking Status (% Smoker) | 54.8 | 72.2 | 76.0 | | X^2^=3.15, *p*=0.21, ns |
| IQ | 106.9 (9.3) | 106.1 (7.9) | 99.6 (11.3) | | F=4.23, df 2,71, *p*=0.018 H>P |
| Years of Education | 13.6 (2.8) | 12.6 (3.0) | 10.9 (1.9) | | F=7.63, df 2,71, *p*=0.001 H>P |
| Edinburgh Handedness | 43.3 (60.4) | 59.5 (57.0) | 59.9 (64.6) | | F=0.65, df 2,71, *p*=0.65 ns |
| Months Abstinence | - | 14.8 (19.8) | 21.1 (35.2) | | t=-0.67, df 41, *p*=0.5 ns |

Table S2. Demographics of participants included in the Go/No-go task analysis. One way ANOVA and Sidak post-hoc tests were used to compare continuous demographic variables. Chi-squared tests were used for categorical variables. A 2-sample t-test was used to test for differences in months of abstinence between abstinent alcohol and poly-drug dependent groups. Means and standard deviation (in brackets) shown for continuous variables. Abstinence from the main drug of dependence was used for the poly-drug group.

Participants

Two participants in the PD group admitted use of cocaine on one occasion approximately 48 hours before testing and subsequently tested positive. Participants showed no signs of intoxication (heart rate and blood pressure within normal range) or withdrawal as assessed with the Cocaine Selective Severity Assessment, therefore both participants were permitted to complete the testing session.

*Reasons for participant exclusion*

Five people were excluded from the Monetary Incentive Delay (MID) task analysis: 2 healthy control (HC), 1 poly-drug (PD), and 2 alcohol dependent (AD) participants. One was excluded for excessive head movement (>10% of volumes having a scan-to-scan displacement >3mm), 1 for signal dropout, 2 for failing to perform the task as instructed (keeping their finger on the response pad button and/or repeatedly pressing the button during the anticipation phase, effectively “cheating” to increase winnings) and 1 for scanner failure. A total of 83 participants were entered into the MID task analysis. For the Go/No-Go (GNG) task, 14 exclusions were made (4 HC, 8 PD, 2 AD). Eleven of these were participants either falling asleep and/or not engaging with the task (defined as a baseline condition accuracy of <80%), 1 for excessive movement, 1 for signal drop out and 1 for scanner failure. A total of 74 participants were therefore included into the GNG analysis.

**Methods**

**Task Description**

*MID*

Participants could win or lose money depending upon how quickly they reacted to a target stimulus. The task contained win (win £0.50), loss (lose £0.50) and neutral (neither win nor lose) trials. Participants were informed of the type of trial they were about to perform by cues appearing on the screen for 1 second. There was an anticipation period (2, 3 or 4 seconds) before the target stimulus was presented. The duration of the target stimulus increased or decreased by 10ms depending upon the accuracy of participants (until a minimum duration of 150ms or a maximum duration of 300ms was reached) to obtain an accuracy of approximately 66% for win trials. Participants were informed if they were successful, together with a display of their total winnings, via an “outcome” slide presented for 2 seconds, 500ms after the onset of the target stimulus. The outcome slide was followed by a period of fixation before the next trial began. Two runs of the task were carried out with each run 7 minutes 12 seconds in duration. Each run contained 18 win and neutral trials and 6 loss trials (loss trials were included to increase the salience of win trials). In total, the task comprised of 36 win, 36 neutral and 12 loss trials.

*GNG*

The GNG task was an event-related task adapted from Garavan *et al* 2002, consisting of a series of letter Xs and letter Ys (presented for 900ms followed by a 100ms inter-stimulus interval). Participants were instructed to respond as fast as they could to each letter presented (go trial) except when the letter was the same as the previous letter (no-go trial). Two runs of the task were carried out, with each run 4 minutes 22 seconds in duration. Each run contained 220 go trials and 30 no-go trials. In total, the task comprised of 540 go trials and 60 no-go trials

Both tasks were programmed using E-Prime (version 2.0.8.90).

**MRI Data Acquisition**

Data were acquired across 3 different centres with London and Cambridge operating 3T Siemens Trio systems and Manchester operating a 3T Philips Achieva. At all centres, T1-weighted volumes were acquired using a magnetization-prepared rapid gradient echo (MPRAGE) sequence. Functional imaging was performed using a multi-echo gradient echoplanar imaging (EPI) sequence (TR = 2000 ms, TE = 13 & 31 ms, flip angle = 80°, field of view = 225 mm, image matrix = 64 x 64) with an in-plane resolution of 3.516 x 3.516 mm and a slice thickness of 3.000 mm. In London and Cambridge, 36 abutting oblique axial slices were collected in an ascending manner at an angle of around 30° to the anterior (AC) and posterior commissure (PC) line for each volume whereas 34 slices were collected in Manchester. This resulted in slightly less than whole brain coverage, with the most superior 9 mm not being imaged in most participants.

**Preprocessing**

The data were preprocessed using SPM12b. Both runs of the both tasks were preprocessed separately. The data were realigned using the 1^st^ image as reference. The T1 structural image was coregistered with the mean realigned functional image to ensure the structural and functional images were in the same space, before segmentation and normalisation of the structural image into a standard stereotatic space was carried out using the unified segmentation approach . These normalisation parameters were then applied to the functional images such that all functional images were warped into a standard space. Smoothing was carried out using an isotropic Gaussian smoothing kernel with a FWHM of 8mm for the GNG task and 7mm for the MID task given reports that smaller smoothing kernels may be more appropriate for reward tasks with subcortical regions of interest of a small volume . Detection of movement outliers (>3mm) was carried out using the Artifact Detection Toolbox (ART) (https://www.nitrc.org/projects/artifact_detect/).

**1^st^ level analysis**

MID

Analysis focused on the ‘cue and anticipation’ phase of the task and was modelled as a mini-block beginning at the cue onset and ending at the onset of the target stimulus. Mini-blocks were convolved with the haemodynamic response function and had a duration of either 3, 4 or 5 seconds (1 second of cue + anticipation phase which was either 2, 3 or 4 seconds). The contrast of interest explored is the average of the ‘reward cue anticipation’ compared with ‘neutral cue anticipation’ across both runs. The outcome phase was not investigated due to concerns over contamination from signals of the anticipation phase. The task was not powered to examine loss trial anticipation (loss trials were only included to increase the salience of win trials) and therefore loss anticipation was not investigated. Realignment parameters and movement outliers generated by ART were added to the 1^st^ level model as nuisance regressors. A high pass filter of 60 seconds was used. No global normalisation or temporal derivatives were implemented. Serial correlations were accounted for using AR(1) modelling.

GNG

Successful inhibitions of nogo trials (“stops”) and unsuccessful nogos (“errors”) were modelled against an implicit baseline of go trials. Both stops and errors were convolved with the haemodynamic response function. Stops that were preceded by a go trial that also did not have a response were considered “fake inhibitions” as it is likely these apparently successful inhibitions were due to lapses in attention rather than active inhibition. These were modelled separately as conditions of no-interest. Realignment parameters and movement outliers were included as nuisance regressors. A high-pass filter cut-off of period above 120 seconds was applied. No global normalisation or temporal derivatives were implemented. Serial correlations were accounted for using AR(1) modelling. The task was powered to look at “stops” rather than “errors” therefore only the “stops>go” contrast is explored further.

**Regions of Interest Definition (ROI)**

For the MID task, both the ventral striatum and ventral pallidum ROIs were defined according to the guidelines set out in Tziortzi et al, 2011 enabling drug effects to be investigated in relation to D3 receptor density. Both the ventral pallidum and ventral striatal ROIs were hand drawn onto the Ch2better template within MRIcron (Rorden, [www.mricron.com](http://www.mricron.com/)). The substantia nigra was defined on the PET image in Tziortzi et al., 2011 therefore the guidelines for definition cannot be applied here. A substantia nigra mask was therefore taken from the Hammersmith probabilistic atlas© Copyright Imperial College of Science, Technology and Medicine 2007, (Hammers et al 2008). Available from www.brain-development.org.

In contrast to the subcortical ROIs for the MID task, masks for the ROIs for the GNG task (inferior frontal gyrus and anterior cingulate cortex) are supplied within the neuromorphometrics atlas within SPM12 ([www.neuromorphometrics.com](http://www.neuromorphometrics.com/)), therefore this atlas was used to define our GNG regions of interest (the interior frontal gyrus mask was comprised of the opercularis, triangularis and orbitalis regions).

Finally, to ensure ROIs included only task relevant regions, only voxels that were activated in the effect of task 1 sample t-test (contrast of interest for each task, from all participants for both placebo and GSK598809 conditions, thresholded with a whole-brain voxelwise, Family Wise Error (FWE) corrected threshold of *p*<0.05) were included in the final ROIs for each task.

**2^nd^ Level Analyses**

ROI analysis

Mean contrast estimates were extracted from each ROI using the “Easy ROI” SPM toolbox <http://www.sbirc.ed.ac.uk/LCL/LCL_M1.html>) and entered into mixed ANOVAs in SPSS containing group at the between subject factor (HC, AD and PD) and drug session (placebo/GSK598809) as the within-subject factor. Age was included as a mean adjusted covariate in all analyses. For the MID task, right and left ROIs were averaged as there is no compelling evidence for lateralization of reward processing. For GNG, left and right IFG were analysed separately due to the hypothesised right-sided lateralization of this task . The proportions of drug dependent participants to control participants were matched at each site as determined by non-significant Chi-squared tests. A Bonferroni correction for the 3 regions investigated for each task was applied, therefore significance was set at *p*<0.017. Main effects and interactions were explored. Post-hoc t-tests were used to determine the direction of observed ANOVA effects.

Whole-Brain Investigations

As fMRI packages only produce one error term during statistical analysis whole-brain 2^nd^ level analysis were carried out using the toolbox “Mulitivariate Repeated Measures” that correctly partitions within- and between-subject error terms . Mixed ANOVAs were specified containing group as the between subject factor (HC, AD and PD) and drug session as a within-subject factor (Placebo and GSK598809). Age was entered as a covariate. Statistical significance was determined using voxel-level permutation testing. 5000 permutations were carried out for each contrast of interest (main effect of drug, main effect of group and drug by group interaction). The Wilks’ Lamda test statistic was used. Regions were reported as significant if they survived a whole-brain Family Wise Error corrected threshold of *p*<0.05.

**Correlations analyses**

*Brain Activation and Task performance*

Investigations were carried out to determine whether there was any relationship between task performance and ROI brain response under normative conditions. For the MID task, correlations were carried out between each ROI and win-neutral reaction time under the placebo condition. For the GNG task, correlations were carried out between each ROI and % nogo accuracy under the placebo condition. Correlations were carried out in all participants except one participant who did not respond in time for neutral trials of the MID task. Table S3 shows the results of these analyses.

*Associations between reward and impulsivity*

Correlational analyses were carried out to determine whether ventral striatal (VS) and inferior frontal gyrus (R IFG) activation were associated with impulsivity as measured by the Barratt Impulsiveness Scale-11 and/or reward sensitivity as measured by the reward responsiveness subscale of the BIS/BAS . Furthermore, correlational analyses were additionally carried out between MID VS activation and GNG performance (% nogo accuracy) and GNG R IFG activity and MID performance (win-neutral reaction time). To determine whether MID VS blunting in the MID task was associated with GNG R IFG hyperactivation, correlational analyses were carried out between VS activation and R IFG activation. Correlations were carried out in all participants using placebo session data to determine relationships under normative conditions. Table S4 shows the results of these analyses.

*Length of abstinence*

To investigate whether there was any evidence of recovery of brain activation with abstinence, correlational analyses were carried out between length of abstinence (from the main drug of dependence) in the AD and PD groups and brain activation in each ROI. The analysis was carried out in the dependent groups separately using the placebo data. Table S5 shows the results of these analyses.

*Associations between drug effect and performance*

To investigate whether the increased ventral pallidum (VP) win-neutral activation under GSK598809 relative to placebo was associated with a change in MID performance (change in win-neutral reaction time), a correlational analysis was carried out between GSK598809 VP activation - Placebo VP activation, and GSK598809 reaction time– placebo reaction time.

Table S6 shows the results of this analysis.

All correlational analyses were calculated using Pearson’s-product moment correlation coefficient.


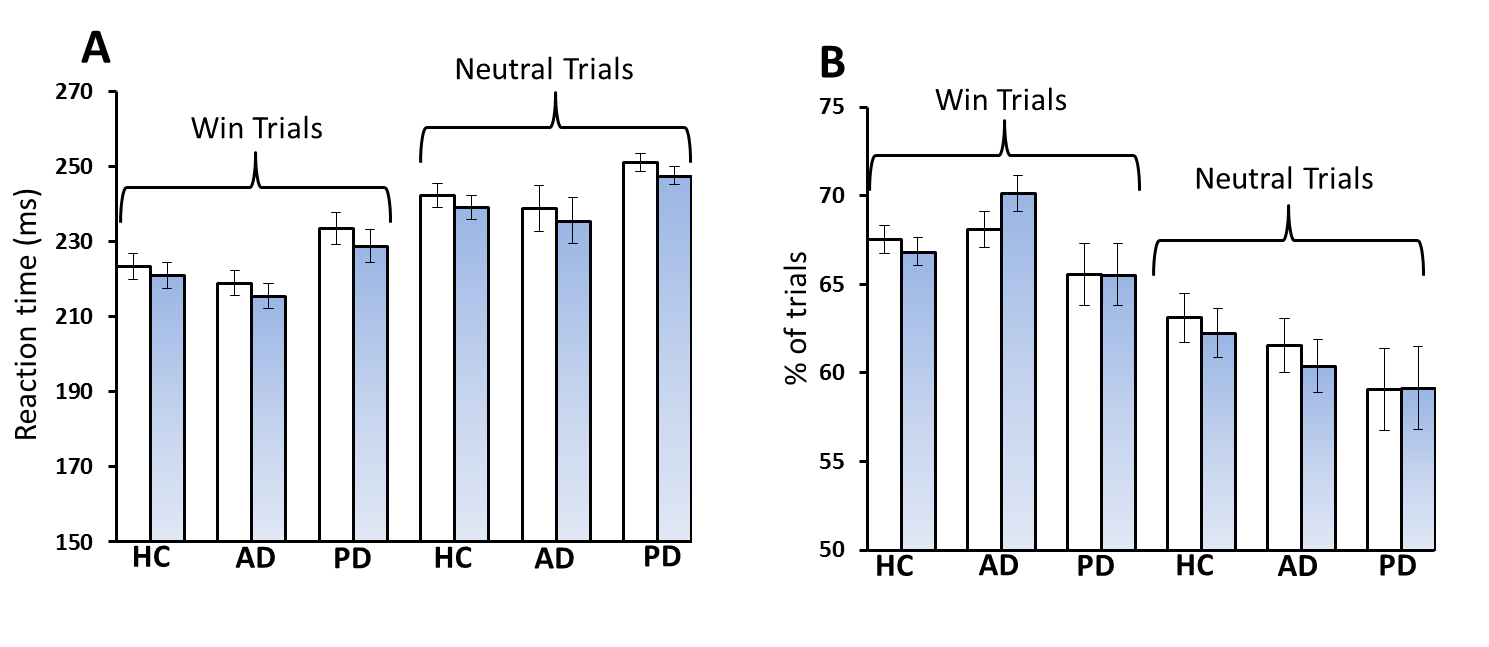


Figure S1 Behavioural results for the MID task. **A)** Reaction time for responses to the target stimulus for both win and neutral trials in the healthy control group (HC), abstinent alcohol dependent group (AD) and the poly-drug dependent group (PD) **B)** % of win and neutral trials where participants responded in time for target stimulus (hits). White = Placebo session, blue = GSK598809 session. Error bars demonstrate the within-subject standard error of the mean appropriate for demonstrating drug effects rather than group effects.


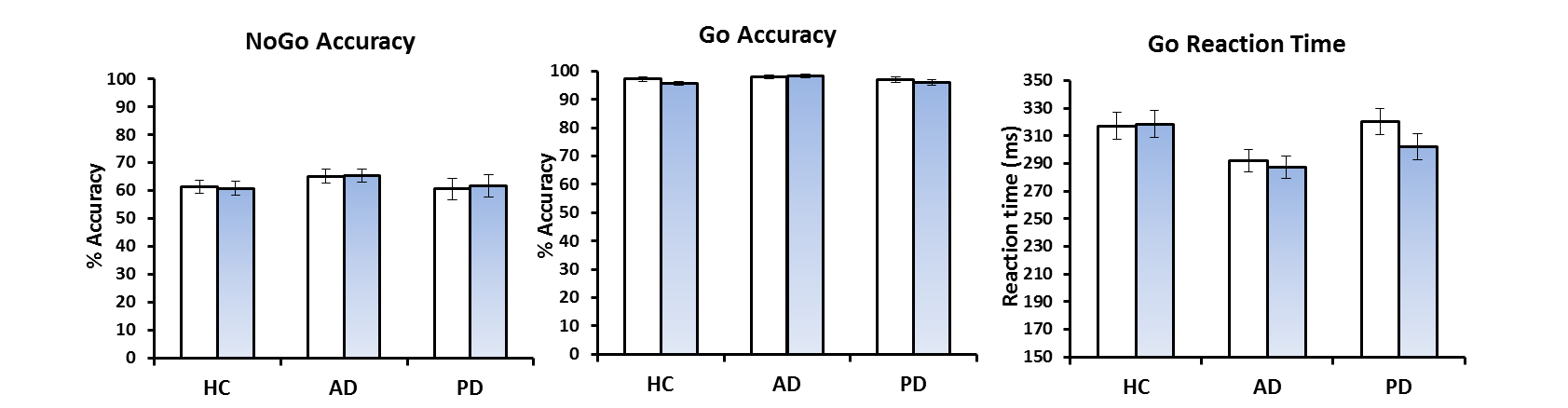


Figure S2. GNG Behavioural Results. White = Placebo session, blue = GSK598809 session. Error bars show within-subject standard error of the mean appropriate for demonstrating drug rather than group effects.


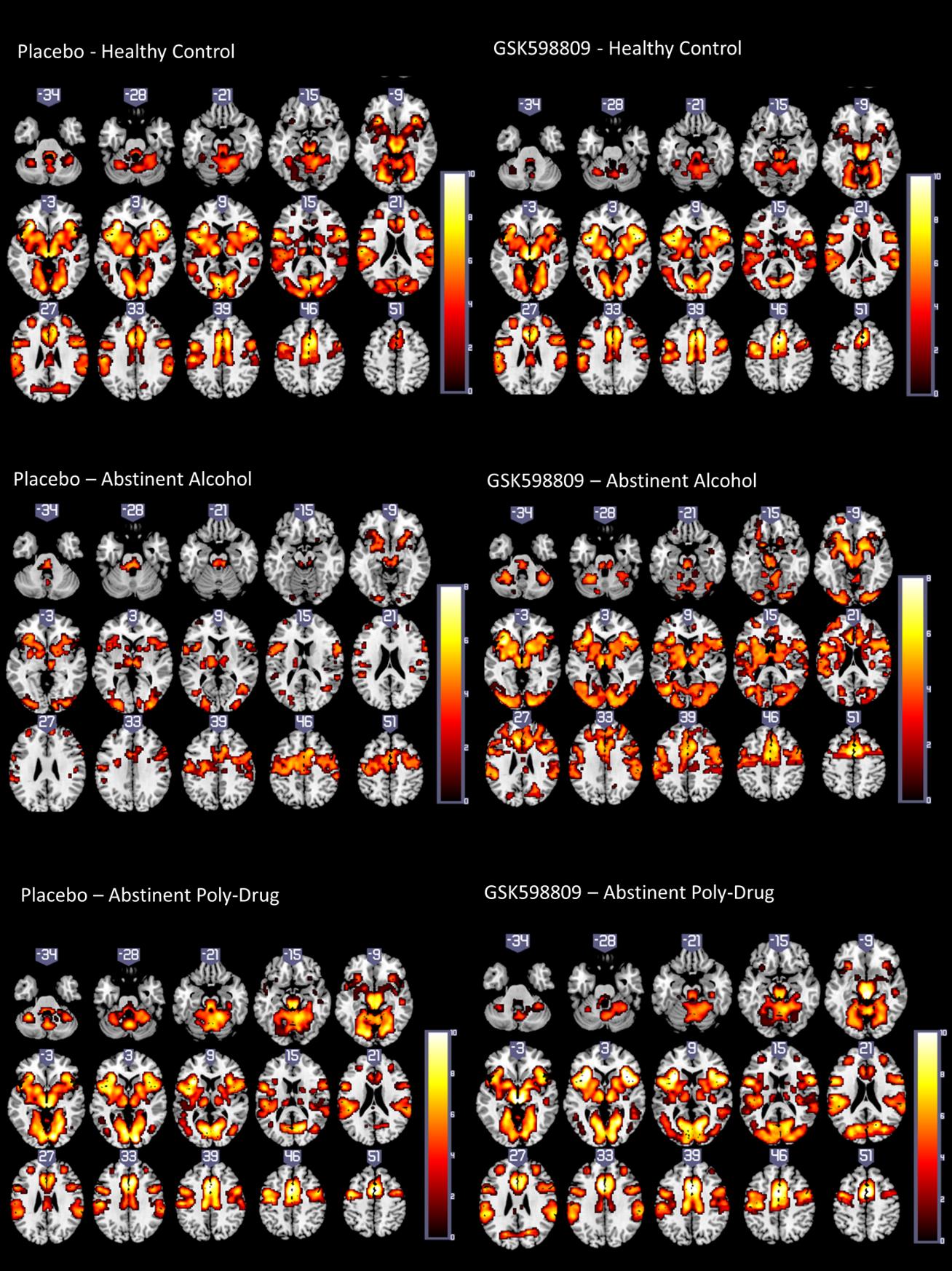


Figure S3 MID activation maps. Images show “reward-neutral anticipation” activation for both the placebo and the GSK598809 session for the heathy control group (n=33), the abstinent alcohol dependent group (n=18) and the abstinent poly-drug dependent group (n=32). The colour bar to the right of each montage shows the t-value. Image thresholded at p<0.001 uncorrected for illustrative purposes.


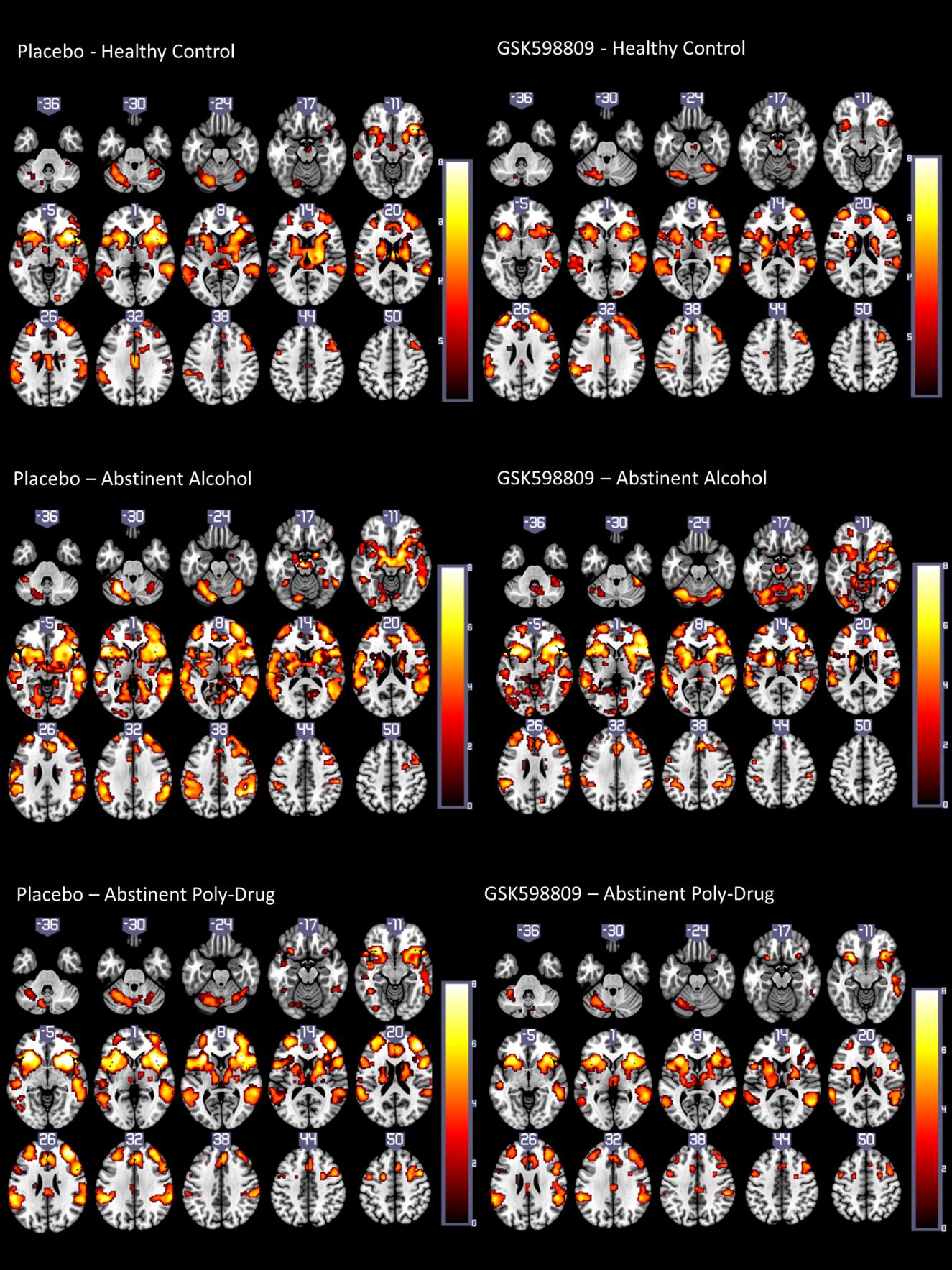


Figure S4 GNG activation maps. Images show “stops-go” activation for both the placebo and the GSK598809 session for the heathy control group (n=31), the abstinent alcohol dependent group (n=18) and the abstinent poly-drug dependent group (n=25). The colour bar to the right of each montage shows the t-value. Image thresholded at p<0.001 uncorrected for illustrative purposes


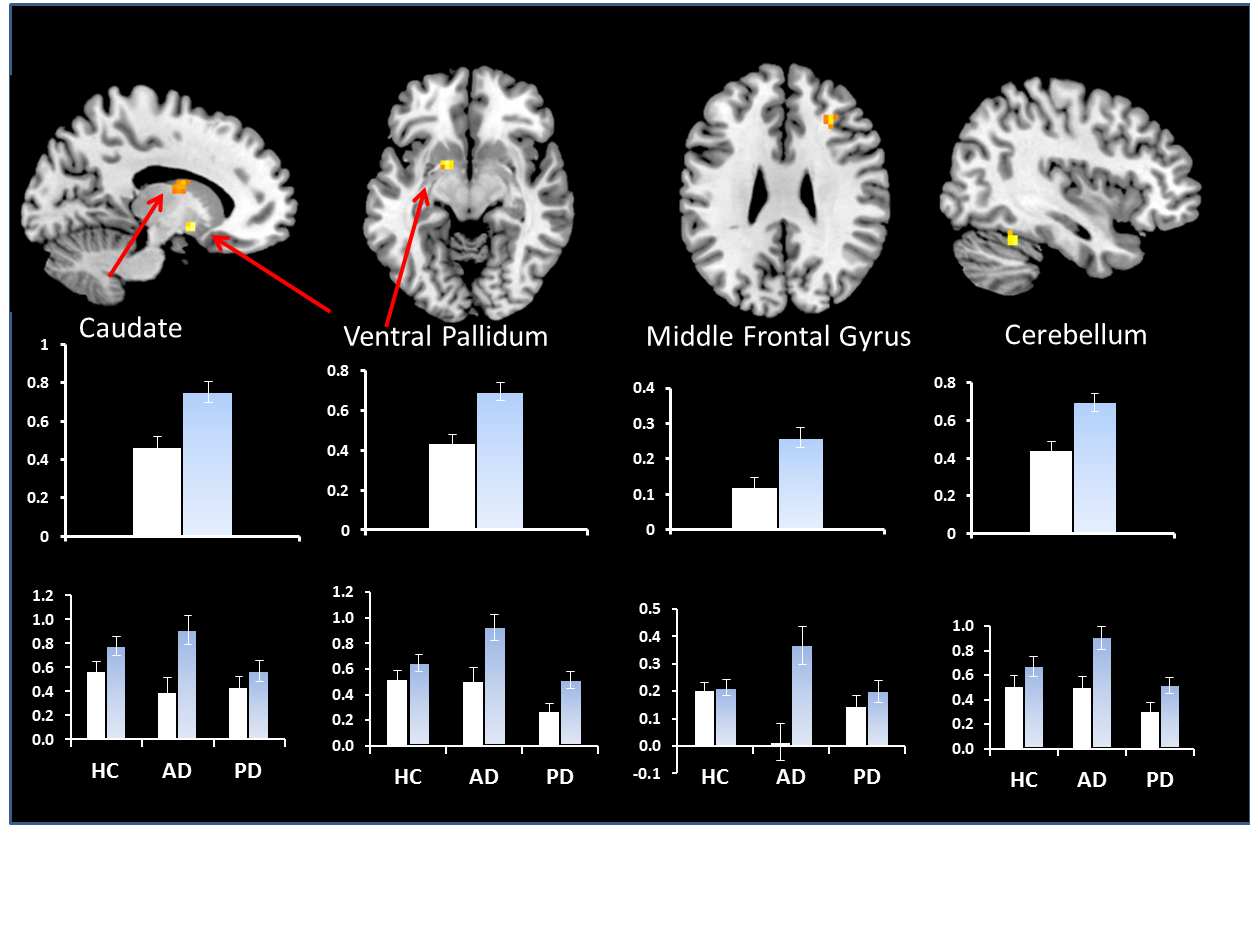


Figure S5. Results from MID whole- brain analysis. Top histograms demonstrate the main effect of drug. Bottom histograms demonstrate the effects within each each group separately. Image thresholded at *p_fwe_*<0.05.


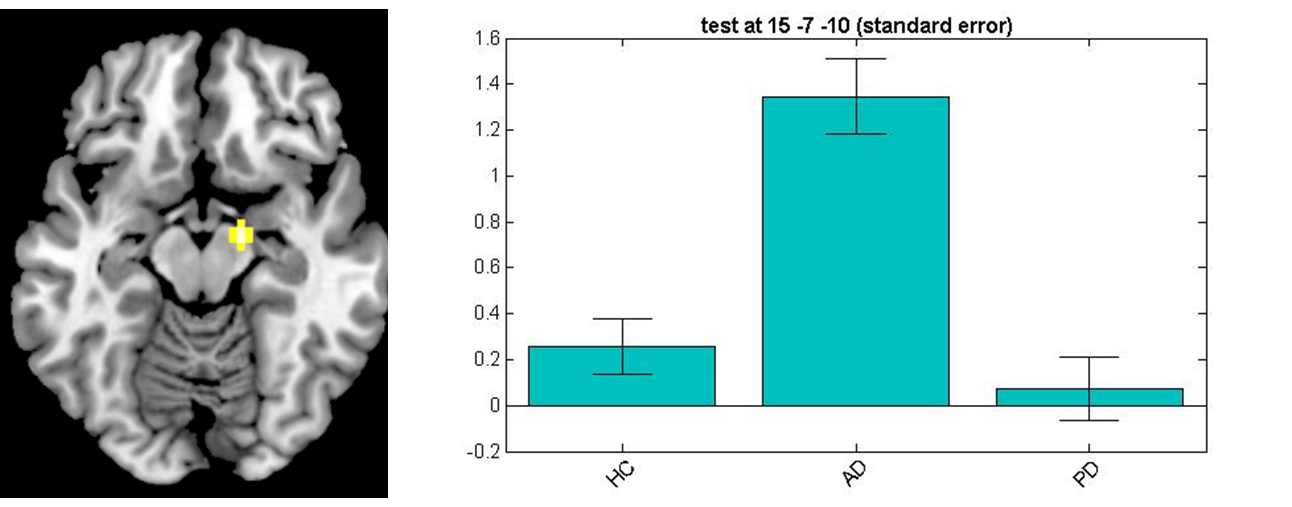


Figure S6. Main effect of group from the whole brain comparison for the GNG task. Image thresholded at *p_fwe_*<0.05.

**MID ROI investigation separated by Primary Drug of Dependence**

The original aim of the ICCAM study was to recruit three distinct groups of alcohol, cocaine and opiate addicts based on dependence on one main substance. However, numerous participants demonstrated co-dependence and were therefore grouped into a “poly-drug” dependent category. A “primary” drug of dependence was however identified for each participant by addiction psychiatrists at each research site based upon the clinical interview and drug timeline follow back .

Reward-Neutral reaction time was investigated with mixed ANOVAs, grouped according to primary dependence (except for HC group as they were not dependent upon any drug except nicotine) and with drug session as the within subject factor. Mixed ANOVAs were also carried out as described for the “ROI analysis” section except participants were grouped according to primary drug of dependence. 26 participants reported primary dependence upon alcohol, 17 upon opiate but only 7 on cocaine. Due to the low number within the cocaine group, analysis focused upon healthy control, primary alcohol dependence (pAD) and primary opiate dependence (pOD). No significant group difference was found for age, gender, IQ or smoking status. There was an overall significance difference for years of education (healthy controls > primary opiate dependence).

As experiments were exploratory in nature, no Bonferroni corrections were applied.

**Results**

No significant main effects or interactions were found for performance.

As with the main analysis, a significant effect in the ventral striatum was found (*p*=0.020). Here however, a drug x group interaction emerged (*p*=0.047). No main effect of group was found. From figure S7 it can be seen that GSK598809 normalises VS response in the primary alcohol dependent group only.

Within the ventral pallidum, there is still a significant main effect of drug (p<0.001) again with a drug by group interaction (*p*=0.017). Again the effects appear to be due to GSK598809 increasing reward-neutral response primarily within the primary AD dependent group with limited effects of GSK598809 in the HC group and the primary opiate dependent group (figure S7). A trend for a main effect of group was found (*p*=0.059) that appeared to be driven by differences between the AD and OD group.

Within the substantia nigra there was an overall significant effect of drug with GSK598809 *(p*=0.008) enhancing reward-neutral BOLD response. No interaction or group effects were found.


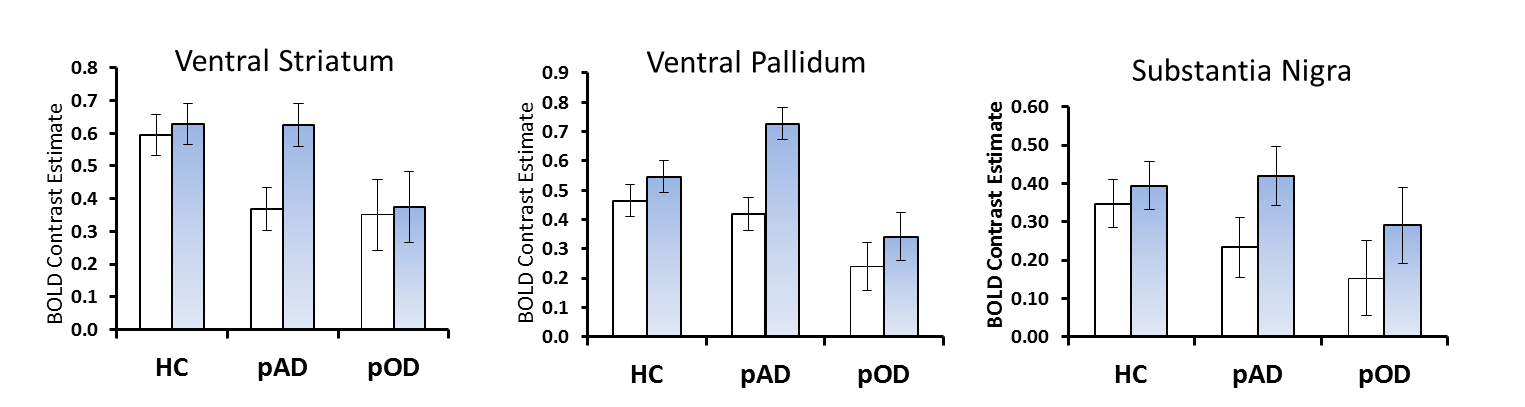


Figure S7. BOLD contrast estimates within regions of interest in groups separated by primary drug of dependence. White bars=placebo session, blue bars=GSK598809 session. HC=healthy control, pAD=primary alcohol dependence, pOD=primary opiate dependence.

**Correlation Tables**

Table S3. MID and GNG task ROI brain activation correlation with task performance.

Table S4. Reward-Impulsivity Variable Correlations

Table S5. MID and GNG task ROI brain activation correlation with duration of abstinence in AD and PD groups

Table S6. Correlation between change in ventral pallidum brain activation and win-neutral reaction time across the GSK598809 and placebo sessions.

**References**

Aron AR, Fletcher PC, Bullmore ET, Sahakian BJ, Robbins TW (2003). Stop-signal inhibition disrupted by damage to right inferior frontal gyrus in humans. *Nature neuroscience* **6**(2): 115-116.

Ashburner J, Friston KJ (2005). Unified segmentation. *NeuroImage* **26**(3): 839-851.

Carver CL, White TL (1994). Behavioral inhibition, behavioral activation, and affective responses to impending reward and punishment: The BIS/BAS Scales. *Journal of personality and social psychology* **67**(2): 14.

Cousineau D, O’Brien F (2014). Error bars in within-subject designs: a comment on Baguley (2012). *Behavior Research Methods* **46**(4): 1149-1151.

Garavan H, Ross TJ, Murphy K, Roche RA, Stein EA (2002). Dissociable executive functions in the dynamic control of behavior: inhibition, error detection, and correction. *NeuroImage* **17**(4): 1820-1829.

McFarquhar M, McKie S, Emsley R, Suckling J, Elliott R, Williams S (2016). Multivariate and repeated measures (MRM): A new toolbox for dependent and multimodal group-level neuroimaging data. *NeuroImage* **132**: 373-389.

McLaren DG, Schultz AP, Locascio JJ, Sperling RA (2011). Repeated-measures designs overestimate between-subject effects in fMRI packages using one error term. *17th Annual Meeting of Organization for Human Brain Mapping.* : Quebec City, Canada.

Paterson LM, Flechais RS, Murphy A, Reed LJ, Abbott S, Boyapati V*, et al* (2015). The Imperial College Cambridge Manchester (ICCAM) platform study: An experimental medicine platform for evaluating new drugs for relapse prevention in addiction. Part A: Study description. *J Psychopharmacol* **29**(9): 943-960.

Patton JH, Stanford MS, Barratt ES (1995). Factor structure of the Barratt impulsiveness scale. *Journal of clinical psychology* **51**(6): 768-774.

Sacchet MD, Knutson B (2013). Spatial smoothing systematically biases the localization of reward-related brain activity. *NeuroImage* **66**: 270-277.

Tziortzi AC, Searle GE, Tzimopoulou S, Salinas C, Beaver JD, Jenkinson M*, et al* (2011). Imaging dopamine receptors in humans with [11C]-(+)-PHNO: dissection of D3 signal and anatomy. *NeuroImage* **54**(1): 264-277.
